# Psychometric properties of the Spanish version of the MOS social support survey among caregivers of patients with advanced illness receiving palliative care

**DOI:** 10.3389/fpsyg.2025.1715994

**Published:** 2025-12-04

**Authors:** Sonia Carreño-Moreno, Mauricio Arias-Rojas, Jennifer Rojas-Reyes

**Affiliations:** 1 Faculty of Nursing, Universidad Nacional de Colombia, Bogotá, Colombia; 2 Faculty of Nursing, Universidad de Antioquia, Medellín, Colombia

**Keywords:** caregivers, social support, psychometrics, palliative care, health care surveys

## Abstract

**Introduction:**

Family-reported outcome measures are essential to assess the impact of illness and care on family caregivers in palliative care (PC). The Medical Outcomes Study Social Support Survey - MOS-SSS is widely used to measure perceived social support. However, the number of validated versions for Spanish-speaking caregivers in PC contexts remains limited. This study aimed to evaluate the psychometric properties of the MOS-SSS in a Colombian sample of family caregivers of patients with advanced illness receiving palliative care.

**Methods:**

A psychometric cross-sectional study was conducted with caregivers of patients with cancer and heart failure receiving PC in two hospitals in Medellín-Colombia. The instrument was administered through structured interviews conducted by trained researchers. Construct validity was assessed using confirmatory and exploratory factor analyses. Internal consistency, stability, convergent and known-groups validity, and sensitivity to change were evaluated. The analyses followed COSMIN guidelines.

**Results:**

A total of 434 family caregivers (mean age = 45.7 ± 14.6 years; 86% female) of patients receiving PC were included. Confirmatory factor analysis supported a four-factor model with acceptable fit indices (CFI = 0.94, TLI = 0.92, RMSEA = 0.07, SRMR = 0.043), although a high covariance was observed between the domains of positive social interaction and affectionate support (*r* = 0.899). An EFA revealed a three-factor model with acceptable fit and theoretical coherence (CFI = 0.94, TLI = 0.920, RMSEA = 0.073, and SRMR = 0.044). Cronbach’s alpha for the total scale was 0.94, and McDonald’s Omega was 0.96. Stability was good to excellent (0.85–0.89). Convergent validity was confirmed using the Quality of Life in Life Threatening Illness-Family Carer Version scale (rho = 0.426, *p* < 0.001), and known-groups validity showed lower scores among sole caregivers. The scale was not sensitive to changes following a brief educational intervention.

**Discussion:**

The Spanish version of the MOS-SSS demonstrated strong psychometric properties for assessing perceived social support in family caregivers within PC. The findings support its use in clinical and research settings in Latin American contexts. Future research should explore the responsiveness of interventions explicitly designed to modify the social support networks of caregivers.

## 1 Introduction

Family-reported outcome measures (FROMs) are essential in the field of palliative care (PC), because by definition, PC services are not limited to the patient but extend to the family as a unit of care (Centeno et al., 2018). A recent report from the World Innovation Summit for Health highlighted the importance of FROMs in promoting family caregivers’ health and well-being (Harding et al., 2024; Lambert et al., 2025). In regions such as Latin America, FROMs should be multidimensional to capture complex and context-specific phenomena associated with advanced illness, and must be validated for the populations in which they are used (Bausewein et al., 2016).

The Medical Outcomes Study Social Support Survey (MOS-SSS) (Sherbourne and Stewart, 1991) was originally developed in 1991 to assess perceived social support among individuals with chronic conditions and has since become a widely used FROM (Dao-Tran et al., 2023). The MOS-SSS has versions for both patients and family caregivers and is designed to evaluate the perceived availability of caregiving-related social support resources. Social support was conceptualized as a functional construct, referring to the perceived availability of emotional, informational, tangible, and social interactional assistance (Sherbourne and Stewart, 1991). The MOS-SSS specifically measures these dimensions through four subscales: emotional/informational, tangible, positive social interaction, and affectionate support. It has been applied in diverse settings, including among caregivers of hospitalized patients, in community contexts, and in healthcare research (Hsu et al., 2019; Sibalija et al., 2020).

Since its development, the MOS-SSS has been translated into 14 languages, allowing for its use across a variety of populations and cultural contexts worldwide (Dao-Tran et al., 2023). Spanish is particularly relevant in Latin America, a region that, along with other low- and middle-income countries worldwide, accounts for an estimated 60–70% of the global need for PC. However, according to the Latin American Atlas of Palliative Care (Pastrana et al., 2020), the provision of these services in low- and middle-income countries in the region ranges from early-stage capacity building to generalized service delivery. This contrasts with high-income countries, where PC is fully integrated into healthcare systems (Clark et al., 2020).

To promote the development of PC in Latin America, access to validated instruments that can assess and monitor the perceived social support of both patients and families is critical. Given that Spanish is the primary language across Latin America, where access to validated tools remains scarce, it is essential to ensure culturally and linguistically appropriate instruments for PC practice and research. Several studies in the region have identified multiple unmet needs among family caregivers and a marked lack of perceived social support (Dittborn et al., 2021), reinforcing the importance of having a culturally validated Spanish version of the MOS-SSS.

Spanish-language adaptations of the MOS-SSS have been reported in Spain (Costa Requena et al., 2007), Mexico (Merino-Soto et al., 2023), and Colombia (Londoño Arredondo, 2012). However, few of these have been tested among family caregivers in PC settings. These adaptations may not fully align with the unique experiences of family caregivers in the context of PC. In the Latin American context, caregivers often face not only limited access to resources and insufficient support from healthcare systems, but also delayed diagnoses of their loved ones’ illnesses (Reid et al., 2021). This context contributes to heightened emotional distress, uncertainty, and increased need for support—not only for the demands of daily care and logistical concerns but also to cope with the patient’s anticipated death. This study aimed to evaluate the psychometric properties of the MOS-SSS in a Colombian sample of family caregivers of patients with advanced illness receiving palliative care.

## 2 Materials and methods

### 2.1 Study design

A psychometric validation study with a longitudinal component was conducted between 2023 and 2024 conducted in Medellín, Colombia.

The methodological guidelines for validation proposed by the COSMIN initiative were followed (Gagnier et al., 2021). Given that the MOS-SSS had already been translated into Spanish and culturally adapted for the Colombian context—with prior evidence of face and content validity in Latin American populations—this study was designed to further evaluate additional psychometric properties, specifically those related to construct validity, criterion validity, internal consistency, test–retest reliability, sensitivity to change, and known-groups validity, to confirm the instrument’s suitability for family caregivers in PC populations in Colombia.

The study was conducted in accordance with the ethical principles of the World Medical Association (2013) and the COSMIN reporting guidelines (Gagnier et al., 2021). Ethical approval was obtained from the Ethics Committee of the university affiliated with one of the authors (File No. CEI-0096-02-2022) and from the ethics committees of the hospitals where data collection took place (File No. CEI-0293-2022). All participants provided written informed consent before participation.

### 2.2 Participants

The participants were informal family caregivers of patients with advanced cancer or heart failure recruited from two hospitals in Medellín. Eligible participants were adults aged > 18 years, fluent in Spanish, identified as the primary caregiver, and cognitively intact (as determined by the Mini-Mental State Examination). Caregivers were included if the patient was receiving palliative treatment for an advanced illness, as confirmed in the medical record. The exclusion criteria included being a hired caregiver, being enrolled in another institutional educational study, or caring for a patient in the active dying phase (Karnofsky Index ≤ 20).

### 2.3 Procedures

Caregivers were identified via daily lists of PC patients in the two hospitals. A trained investigator approached the patients in their rooms and identified the primary caregiver. After confirming eligibility and explaining the study objectives, caregivers who agreed to participate provided written informed consent. Baseline data collection (T1) was conducted in the hospital room or waiting area, with the assistance of a trained research assistant.

**Figure 1:**
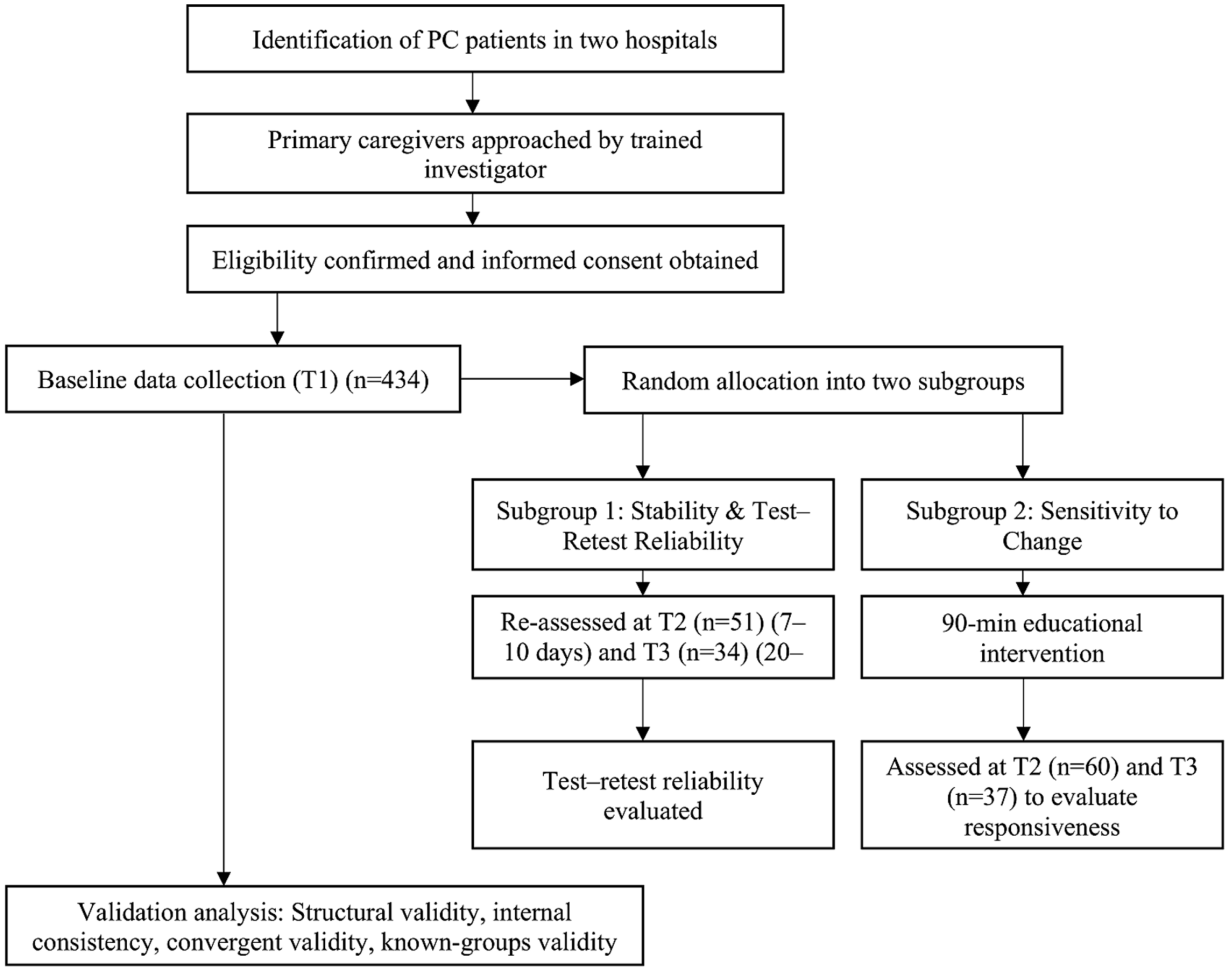
Study flow diagram.

Two subsamples were drawn from the overall sample of participants and randomly allocated to two subgroups using a random number table generated in Excel, ensuring independent datasets for the assessment of both stability and sensitivity to change. In the first subgroup, caregivers whose caregiving context, patient condition, and personal circumstances remained stable were re-assessed at 7–10 days (T2) and again at 20–30 days (T3) to evaluate test–retest reliability. In the second subgroup, caregivers received a 90-min educational intervention on caregiving, self-care, and social support access (Arias-Rojas et al., 2020). This brief educational intervention was implemented to assess the instrument’s responsiveness rather than to evaluate the intervention’s efficacy. Participants in this subgroup also completed the scale at T2 and T3 to determine its sensitivity to change. Follow-up data (T2 and T3) were collected via telephone with the assistance of the same research assistant (Figure 1).

### 2.4 Sample size

The sample size was determined according to the planned psychometric analyses. A target of over 400 participants was established to meet the recommended for confirmatory factor analysis (CFA) according to Kyriazos (2018), and to exceed the minimum of 200 participants suggested by other methodological guidelines (White, 2022). A minimum of 40 participants per subgroup was estimated for the T2 and T3 subgroup analyses to ensure adequate statistical power.

### 2.5 Instruments

All participants completed a demographic questionnaire that included caregiving duration, hours of daily care, and patient functionality (measured using the Karnofsky Performance Scale).

The MOS-SSS was used to assess social support, which evaluates the frequency of perceived support across four domains: emotional/informational support (items 3, 4, 8, 9, 13, 16, 17, 19), tangible support (items 2, 5, 12, 15), positive social interaction (items 7, 11, 14, 18), and affectionate support (items 6, 10, 20). Item 1 is excluded from any subscale, because it is designed to quantify the support network of the person. Responses are scored on a 5-point Likert scale from 1 (“never”) to 5 (“always”), with total scores ranging from 19 to 95; higher scores indicate greater perceived support. This study used the Spanish version adapted for the Colombian context, with previously reported face and content validity, internal consistency (*α* = 0.93), and a four-factor structure consistent with the original instrument (Londoño Arredondo, 2012).

Before data collection, two bilingual palliative-care experts reviewed the Spanish MOS-SSS to confirm semantic and cultural equivalence for Colombian caregivers, followed by brief cognitive interviews with five participants to ensure clarity and contextual appropriateness. No major modifications were required.

Participants also completed the Quality of Life in Life-Threatening Illness–Family Caregiver version (QOLLTI-F) scale, a 16-item instrument designed to assess quality of life from the perspective of PC family caregivers (Cohen et al., 2006). Items are scored from 0 to 10, with higher scores indicating better quality of life. Previous research in Colombia with caregivers of patients with advanced cancer reported an internal consistency of 0.84 and test–retest reliability of 0.60 for this instrument (Arias-Rojas et al., 2023).

### 2.6 Statistical analysis

Data were collected on paper and entered into a Microsoft Excel database. As a trained assistant helped participants complete the forms, no missing data were reported. All statistical analyses were performed using R software. Descriptive statistics were used to summarize the sociodemographic data. The Kolmogorov–Smirnov test was applied to assess the normality of the MOS-SSS, and the results indicated that the distribution did not meet normality assumptions; therefore, nonparametric tests were used in subsequent analyses.

To assess structural validity, a CFA was first conducted to test the original four-factor structure proposed in the MOS-SSS. This approach was chosen to directly verify the factor configuration reported in the original version of the instrument, consistent with the recommendation of the COSMIN initiative (Mokkink et al., 2016) that cross-cultural validations should aim to reproduce equivalent measurement structures to enable international comparability. However, because the initial CFA indicated a suboptimal model fit, an exploratory factor analysis (EFA) was subsequently performed using principal component extraction and Oblimin rotation to examine alternative structures, particularly the potential emergence of a three-factor solution more consistent with the empirical data. Factors were retained based on eigenvalues greater than 1. The total sample was randomly split into two groups: a subsample of 200 participants was used for the EFA, while the remaining 234 participants were included in the CFA to evaluate the model fit of the resulting three-factor structure. Model fit was assessed using the following indices and recommended cut-off values: RMSEA < 0.08, CFI ≥ 0.90, TLI ≥ 0.90, and SRMR < 0.08 (Anunciada et al., 2022).

Internal consistency was assessed using Cronbach’s alpha for the total scale and each subscale. Additionally, McDonald’s omega was also calculated. Acceptable reliability was defined as *α* or *ω* ≥ 0.70; good ≥ 0.80; excellent ≥ 0.90. To test the hypothesized positive correlations between MOS-SSS scores and both QOLLTI-F and patient functionality (Karnofsky score), convergent validity was assessed using Spearman’s rho. Test–retest reliability was evaluated using Intraclass Correlation Coefficients (ICC) for T1–T2 and T2–T3 in the stable subgroup. ICC values were interpreted as poor (< 0.50), moderate (0.50–0.74), good (0.75–0.89), and excellent (≥ 0.90) [Erratum to “A Guideline of Selecting and Reporting Intraclass Correlation Coefficients for Reliability Research” (Koo and Li, 2016)].

The sensitivity to change was assessed in the intervention group using the Wilcoxon signed-rank test, comparing MOS-SSS scores across T1, T2, and T3. Known-groups validity was tested using the Mann–Whitney U test to compare MOS-SSS scores between caregiver groups (sole caregivers vs. those with additional support, and caregivers of patients with cancer vs. heart failure).

## 3 Results

### 3.1 Participants’ sociodemographic characteristics

A total of 434 family caregivers of individuals receiving PC were included in the study, of whom 354 (81.5%) were caring for patients with cancer and 80 (18.5%) for patients with heart failure. For the analysis of sensitivity to change, 60 participants completed the survey at Time 2 (T2) and 37 at Time 3 (T3). In the stability subgroup, 51 caregivers completed T2 and 34 completed T3. The sample comprised predominantly female participants (86%), with a mean age of 45 years. Table 1 presents the detailed sociodemographic characteristics.

**Table 1:** Sociodemographic characteristics of family caregivers.

Characteristics	N/Mean (*N* = 434)	%/SD
Caregiver with a diagnosis		
Yes	192	44.2
No	242	55.8
Age	45.70	14.56
< 50 years	249	57.4
≥ 50 years	185	42.6
Gender		
Male	95	21.9
Female	339	78.1
Marital status		
Married	245	56.5
Single	189	43.5
Education level		
Primary school	94	21.9
Secondary school	145	33.4
Tertiary education	33	7.6
University education	161	37.1
Occupation		
Employee	183	77.5
Unemployed	28	11.9
Caregiver since the patient’s diagnosis		
Yes	399	91.9
No	35	8.1
Sole caregiver		
Yes	163	62.4
No	271	37.6
Relationship with the patient		
Friend	109	25.1
Partner	103	23.7
Children	36	8.3
Parents	186	42.9
Time as a caregiver (months)	27.4	46.5
Hours dedicated to proviging daily care	18.2	6.7
Patient diagnosis		
Cancer	354	81.5
Cardiovascular	80	18.5

### 3.2 Structural validity

A CFA was initially conducted to evaluate the proposed four-factor structure in the original version of the scale. The results showed factor loadings ranging from 0.48 to 0.89 across all items, with a total explained variance of 68.5%. The model fit indices were acceptable: *χ*^2^/df = 3.18, *p* < 0.001, CFI = 0.94, TLI = 0.925, RMSEA = 0.07, and SRMR = 0.043. Examination of inter-factor covariances revealed values between 0.20 and 0.50, indicating moderate correlations. However, a notably high covariance (*r* = 0.899) was observed between the positive social interaction and affectionate support factors, indicating a substantial conceptual overlap between these two factors.

In response to this finding, an EFA was performed using a subsample of 200 participants to identify an alternative structure with reduced factor collinearity. Sampling adequacy was confirmed by a Kaiser-Meyer-Olkin index of 0.938 and significant Bartlett’s test of sphericity (*p* < 0.001). The EFA suggested a three-factor structure with eigenvalues greater than one and a total explained variance of 64.4%. In this model, the items originally assigned to the positive social interaction and affectionate support domains were merged into a single factor.

This three-factor model was evaluated through a CFA using the remaining subsample of 234 participants, which demonstrated acceptable model fit indices: *χ*^2^/df = 3.32 (*p* < 0.001), CFI = 0.94, TLI = 0.920, RMSEA = 0.073, and SRMR = 0.044. Some cross-loadings were observed for items 2, 5, 12, 15, and 20; however, in all cases, the factor theoretically corresponding to each item had the highest loading. Regarding inter-factor covariances, correlations were 0.35 between factor 1 and factor 2, 0.60 between factor 1 and factor 3, and 0.33 between factor 2 and factor 3 (Table 2).

**Table 2:** Factorial analysis of the MOS-SSS.

Item	Communalities	Factor 1 (emotional/informational support)	Factor 2 (tangible support)	Factor 3 (positive social interaction and affectionate support)
2	0.60	0.55	**0.73**	<0.1
3	0.59	**0.74**	0.35	<0.1
4	0.65	**0.76**	0.32	0.43
5	0.52	0.56	**0.79**	<0.1
6	0.58	0.25	<0.1	**0.74**
7	0.62	<0.1	0.21	**0.78**
8	0.41	**0.60**	0.19	0.11
9	0.69	**0.83**	<0.1	0.13
10	0.62	<0.1	<0.1	**0.78**
11	0.68	<0.1	<0.1	**0.84**
12	0.66	0.55	**0.75**	0.33
13	0.53	**0.73**	<0.1	<0.1
14	0.47	0.27	0.23	**0.67**
15	0.52	0.57	**0.76**	<0.1
16	0.59	**0.78**	0.10	<0.1
17	0.55	**0.75**	0.12	<0.1
18	0.58	0.15	<0.1	**0.76**
19	0.62	**0.79**	<0.1	0.26
20	0.51	0.60	<0.1	**0.67**
Eigenvalue	-	8.98	1.78	1.46
% Variance explained	-	47.31	9.38	7.70

Bold values indicates greater factorial weight.

### 3.3 Internal consistency

Cronbach’s alpha for the total MOS-SSS scale was 0.94, indicating excellent internal consistency. The average inter-item correlation ranged between 0.43 and 0.45. For the three factors derived from the factorial structure, Cronbach’s alpha values ranged from 0.84 to 0.90 (Table 3). McDonald’s omega also supported the scale’s reliability, with a total omega of 0.96 and a hierarchical omega of 0.76.

**Table 3:** Internal consistency and test–retest reliability of the MOS-SSS.

Domain	No. Items	Internal consistency	Test–retest reliability	
		Cronbach’s alpha	T1 vs. T2	T2 vs. T3
Emotional/informational support	8	0.90	0.89	0.84
Tangible support	4	0.84	0.80	0.76
Positive social interaction and affectionate support	7	0.89	0.80	0.89
Total MOS-SSS	19	0.94	0.89	0.85

### 3.4 Convergent validity

Spearman’s correlation analyses showed a moderate positive correlation between the total MOS-SSS score and the QOLLTI-F (rho = 0.426, *p* < 0.001), supporting convergent validity. However, no significant correlation was found between perceived social support and patient functional status (rho = −0.043, *p* = 0.374).

### 3.5 Test–retest reliability

The ICC for average measures on the total MOS-SSS score was 0.89 between T1 and T2, and 0.85 between T2 and T3 (*p* < 0.001). Domain-specific ICC values ranged from 0.76 to 0.89 (*p* < 0.001), indicating good to excellent stability over time (Table 3).

### 3.6 Sensitivity to change

No statistically significant differences were observed between T1 and T2 (median scores: 79 vs. 75.5; *p* = 0.64) or between T2 and T3 (75.5 vs. 79*; p* = 0.66).

### 3.7 Validity of known groups

A statistically significant difference was observed in median scores between sole caregivers and caregivers with support, with scores of 74 (*n* = 163) and 83 (*n* = 271) respectively (*p* < 0.001). In contrast, no significant difference was found between the median scores of caregivers of patients with cancer and those caring for patients with heart failure (80 vs. 80; *p* = 0.89).

## 4 Discussion

In this study, the CFA supported the MOS-SSS scale’s original four-factor structure; however, a high covariance between the positive social interaction and affectionate support domains suggested substantial overlap. Consequently, an EFA was conducted, revealing a three-factor structure with acceptable fit indices, in which these two highly correlated domains merged into a single factor. This convergence likely reflects the emotionally demanding nature of PC, where caregivers’ experiences tend to blur the boundaries between positive social interaction and affectionate support. Frequent, close, or warm social interactions are often accompanied by expressions of affection, acceptance, and emotional containment, which may lead to an empirical overlap between the two constructs (Lapa et al., 2025; Ribera-Asensi et al., 2025). Social relationships in Latin American socio-affective contexts are deeply embedded in the values of relational warmth, reciprocity, and collective caregiving. Within these cultural frameworks, caregiving and emotional expression are intertwined—affection is often conveyed through shared activities, companionship, and mutual support. Consequently, positive social interaction is perceived not only as a behavioral exchange but as an intrinsic manifestation of affectionate support (McAndrew et al., 2025), reflecting the collective and emotionally expressive nature of Latin American family and community networks.

The complexity of the four-factor structure has also been reported in the Spanish validation with cancer patients, where the positive social interaction domain was merged into emotional/informational support (Costa Requena et al., 2007). Similarly, the findings of Londoño Arredondo (2012) combined items from affectionate support and positive social interaction into a single factor in a healthy population.

The analysis of internal consistency was satisfactory, with a high Cronbach’s alpha for the total scale (*α* = 0.94), very close to that reported in the original study (*α* = 0.97) (Sherbourne and Stewart, 1991), and other validated versions in caregivers of cancer patients in Mandarin (*α* = 0.97) (Shyu et al., 2006), in English among African American caregivers of children with asthma (*α* = 0.97) (Margolis et al., 2019), and in Portuguese among caregivers of individuals with disabilities (*α* = 0.96) (Moraes et al., 2024).

The correlation between the MOS-SSS and the QOLLTI-F confirmed convergent validity. However, such validity was not established with the Karnofsky patient functionality scale. One study has described a relationship between social support and functional status, suggesting that as the disease progresses and associated variables such as symptom burden, increase, structural social support is more prevalent (Nausheen et al., 2009).

Test–retest reliability was adequate in the subgroup with no clinical changes in either the participant or the patient. These findings indicate that, the MOS-SSS scale yields stable scores over time with good to excellent reliability in the absence of real changes. This result is consistent with previous validations in China (Yu et al., 2004), (ICC = 0.84 after 2 weeks) and Portugal (ICC = 0.98) (Alonso Fachado et al., 2007). This has important implications, because the differences observed in longitudinal measurements using the MOS-SSS are likely to reflect true changes in perceived social support rather than measurement error—thus supporting its use for follow-up and evaluation of the impact of interventions.

Regarding sensitivity to change, no increase in perceived social support scores was observed in the subgroup that received the nursing intervention. The MOS-SSS scale assesses individuals’ perceptions of social support, which may remain stable over short periods or when interventions do not directly target support networks. In this study, the intervention was primarily educational and aimed to improve caregiving knowledge, skills, and self-efficacy rather than to modify the structure or availability of social-support relationships. Consequently, the absence of change in MOS-SSS scores likely reflects the intervention’s scope rather than a limitation of the instrument itself. The responsiveness of the MOS-SSS in interventions specifically designed to mobilize or strengthen social networks, such as those involving family engagement, peer support, or community-based components, should be explored in future studies (Vandepitte et al., 2016; Küçükgüçlü et al., 2018; Yuan et al., 2025). Another possible explanation is that the follow-up period was too short to allow for changes in support networks or relatively stable perceptions.

Finally, this study partially confirmed known-groups validity. Statistically significant differences were found between caregivers who reported being the sole caregiver and those who had support. Sole caregivers reported lower median scores of perceived social support, which aligns with the theoretical expectation that limited support networks are associated with lower perceived emotional, instrumental, and informational support (Ribé et al., 2018). In contrast, the scale did not distinguish between caregivers of patients with different chronic illnesses in PC (i.e., cancer vs. heart failure). Our findings, along with those of Leung et al. (2020), suggest that perceived social support in PC may depend more on family involvement and care organization than on the patient’s specific diagnosis.

The validated Spanish MOS-SSS offers a reliable, culturally relevant tool to assess perceived social support among family caregivers in PC. Clinically, it can help identify caregivers at risk of low support and guide targeted nursing or psychosocial interventions. In research, it enables cross-cultural comparisons and longitudinal evaluations of caregiver outcomes, supporting evidence-based strategies to enhance social support within PC contexts in Colombia and Latin America.

### 4.1 Strengths and limitations

This study has several strengths and limitations. Among its main strengths is the sample size, which provided sufficient participants to conduct robust analyses such as CFA. In addition, the use of two longitudinal measurements across different subgroups allowed for a better understanding of the scale’s performance over time.

Regarding the study’s limitations, the sample was restricted to caregivers of hospitalized patients—a context in which the patient’s unstable condition often mobilizes additional resources and support systems. This may have led caregivers to report higher levels of perceived social support than they might have experienced in routine home-care situations. Although the initial sample size was calculated to meet the requirements for CFA with over 400 participants, the lack of adequate model fit prompted the division of the sample into two subsamples (200 and 234 participants) for exploratory and confirmatory analyses, respectively. Although this decision may have slightly affected some fit indices that are sensitive to sample size, both analyses met the recommended participant-to-item ratio of at least 10:1 (White, 2022). Additionally, the sensitivity-to-change analysis included only 50 participants, which may have limited the statistical power to detect significant effects. Finally, because the study was conducted in Spanish with caregivers of PC patients in Colombia, the findings should be interpreted with caution when generalizing to other Spanish-speaking populations in Latin America, where linguistic and cultural variations may influence the perception of social support.

## 5 Conclusion

This study identified the MOS-SSS scale as a valid and reliable tool for measuring social support among family caregivers of patients receiving PC. A three-factor structure with acceptable model fit indices, good internal consistency and test–retest reliability. The scale demonstrated convergent validity through its correlation with a caregiver quality of life measure and known-groups validity by distinguishing between caregivers with and without additional support. However, in this sample, the scale did not demonstrate sensitivity to change. The validation of the MOS-SSS in the Latin American Spanish-speaking PC context represents a valuable contribution to the field, supporting its use by healthcare professionals in both clinical practice and research.

## Data Availability

The datasets presented in this study can be found in online repositories. The names of the repository/repositories and accession number(s) can be found at Mendeley Data, 10.17632/nd6zxbckdh.1.
